# Factors associated with perceived work-life balance among health workers in Gulu District, Northern Uganda: a health facility-based cross-sectional study

**DOI:** 10.1186/s12889-024-17776-8

**Published:** 2024-01-23

**Authors:** Wilfred Felicity Obina, Juliet Ndibazza, Richard Kabanda, Jonathan Musana, Miisa Nanyingi

**Affiliations:** 1 Faculty of Health Sciences, Uganda Martyrs University, Nkozi, Kampala, P.O Box 5498, Uganda; 2 Department of Health, Catholic Medical Services, Gulu District, Kampala, Uganda

**Keywords:** Perceived work-life balance, Job satisfaction, Burnout, Health workers, Gulu district, Northern Uganda

## Abstract

**Introduction:**

Work-life balance (WLB) plays a significant role in improving career satisfaction and reducing burnout. While health workers’ productivity is considered a key factor in client care, there is limited effort put into examining how health workers perceive the balance of their jobs with family and other societal responsibilities (PWLB), especially in low-income countries where the number of health workers in active patient care is low. The purpose of this study was to assess factors associated with perceived work-life balance (PWLB) among health workers in the rural district of Gulu, Northern Uganda.

**Methods:**

A health facility-based cross-sectional analytical study was conducted. A simple random sampling technique was used to select 384 study participants from the three main hospitals in Gulu District. Data were collected from health workers using a self-administered semi-structured questionnaire and analyzed using STATA version 15. Factors associated with PWLB were determined at a multivariable level using a modified Poisson regression with robust variance with a 95% confidence level and 5% statistical significance. Adjusted prevalent ratios (APR) were used to report the Factors associated with PWLB.

**Results:**

Only 157/384 (40.9%) of the health workers reported a positive perceived work-life balance. Multivariable modified Poisson regression analysis showed positive statistical association with PWLB of a laboratory worker(APR = 1.74, 95% CI: 1.10–2.75); a midwife(APR = 1.82, 95% CI:1.13–2.93) or a nurse (APR = 2.19, 95% CI = 1.45–3.30); working in the inpatient department (APR = 1.97, 95% CI: 1.31–2.96) or laboratory (APR = 2.09, 95%CI: 1.34–3.28); and having a flexible work schedule (APR = 28.32, 95%CI:14.52–55.22); feeling satisfied at work (APR = 1.58; 95% CI:1.17–2.10), and belonging to an association in the community (APR = 32.71, 95% Cl:11.91–89.88). On the other hand, employment tenure of 1–4 years (APR = 0.63,95% CI:0.40–0.99) was negatively associated with perceived work-life balance.

**Conclusion:**

Only four in every 10 health workers experienced a positive perceived work-life balance. The type of profession, duty station, flexibility in work schedule, satisfaction with work, and availability of social support systems, were independent determinants of perceived WLB. Therefore, nurturing a system of reviews of the scheduling of health workers, allowing internal staff rotation, and fostering support systems around the health workers could be beneficial for WLB.

**Supplementary Information:**

The online version contains supplementary material available at 10.1186/s12889-024-17776-8.

## Introduction

Organizations need to take steps to advance the quality of work life of the employees which is advantageous to the organization in the long run. The quality of work-life is directly related to work-life balance [1]. Work-life balance (WLB) defines staff well-being which is an important factor in patient care and satisfaction [2]. This study conceptualizes perceived work-life balance (PWLB) from the individual worker’s perspective as a fair evenness across several work and non-work roles that include time, involvement, and satisfaction balance. Time balance denotes the perceived amount of time devoted by a worker to his professional (job) and personal roles during the 12-hour span [3–5]. The involvement balance represents the proportionate level of emotional involvement of the worker in the professional and personal roles [3, 5], and satisfaction balance is the level of satisfaction felt by the individual as an employee and as a family or societal member [3, 4]. This definition is considered with a relative balance of the priorities human resources in the health sector take amidst the increasing expectations of the clients and the managers of healthcare organizations [4–6]. It should be noted that PWLB is an individual’s subjective appraisal of the accord between his/her work and non-work activities and life generally [6, 8]. A positive PWLB occurs when there is satisfaction at work and employees can carry out activities with minor conflicts in the roles the individuals play in work and personal life [7, 8]. Health workers like employees in other sectors face the challenge of balancing these roles causing stress which affects their productivity.

In the health sector, there has been demonstrable effort in designing strategies to improve workers productivity but not a lot has been put into examining the challenges health workers face in combining paid work and other life roles such as family, community work, leisure and aging [8–10]. Health workers are tasked to meet the expectations of the clients through the provision of quality services as well as balancing the expectations of the family and society. The balance between work, family, and social life is an emerging challenge for both employees and employers. Imbalances may arise not just from demands created by work expectations but also the from the increase or the expectations from society that creates tension among workers [5, 9]. The tension due to the disagreement between work and non-work spheres has increased among the employees, especially those working in the health sector due to frequent occurrences of disease epidemic, with demands which occasionally exceeds the available human resource to cope [6, 7]. Yet, PWLB should indicate the extent to which workers experiences are fulfilled and having his or her needs met in both work and non-work facets of life [10, 11]. When an individual experiences positive PWLB balance; a feeling of well-being, develops resulting into higher productivity(11) [12], reduced absenteeism, and reduced attrition [8].

Quite often during epidemics, as was seen at the peak of COVID-19 pandemic, health workers experience heavy workloads resulting in longer shifts, disruptions in sleep patterns and limited home and social engagement, all of which are manifestations of work-life imbalances [12, 13]. Other effects of work-life imbalances resulting from high burden work such as working in emergency department and working for long hours include burnout mental health problems, and famility conflicts [7, 9–11]. There are a number of factors that have been associated with WLB which include: Individual factors such as age [13, 14], sex [13–16], marital status [13], clinical specialty [13, 16], years of employment, having children [16] and nature of family and parenthood (where the individual has children or not) [16]; Institutional/health facility factors subsequent to type of department where the individual is stationed to work, scheduling of work [15], and other supplementary roles in the department; and community roles such roles in the community services, and social support [15, 17, 18]. The way workers perceive this balance is very important for job satisfaction and their ultimate productivity. Yet, the current trends in the increase in disease epidemics exerts pressure at individual, institutional and community levels on the already constrained health worker force [18–20, 22, 23] especially in low-income countries like Uganda. On the other hand, a negative PWLB, may create adverse effects on the personal and professional lives of health workers [18]. In Uganda, clinical services in a hospital setting are offered by a number of professions which include specialist medical personnel, medical officers, clinical officers, laboratory staff (laboratory assistants, technicians, technologists), radiographers, nurses and midwife.

There is need for identification of context specific factors which relate with PWLB with aim to strengthen the support health workers receive from the managers and policy makers. This study examined the factors related with PWLB with the aim to document practices that may be used implement strategies to improve health workers’ productivity.

## Materials and methods

### Study design and setting

The study was an analytical cross-sectional study which enabled measurement of the factor and outcome variable at the same time. It was conducted in Gulu district, a rural district in Northern Uganda. The study was conducted from March to June, 2021 at three main hospitals: One public regional referral (owned by the government of Uganda), one private not-for-profit (affiliated to the catholic church) and one privately owned hospital. The health system in Uganda comprises of decentralized service delivery system with primary facilities at level IIs and level IIIs offer mainly outpatients care; level IVs and hospitals offer more specialized services and receive the referrals from the lower level [24]. Because the referral system is not fully functional, hospitals carry the primary care patient load as well as the patients requiring specialized care. These three hospitals provide primary care services, specialized health services and referral services from the lower health facilities. These hospitals were considered because of the high volume of patients with a daily average of 400 in-patient admissions and 600 outpatient visits [20, 25]. The high-volume health facilities were purposively selected because burden of the need for work-life balance is felt more health facility setting where there is heavy worker-patient engagement and job demand [26]. We selected all the three hospitals. These hospitals also have fully operational emergency, out-patient, in-patient, and laboratory departments.

### Study population

The study population was the health workers operating under direct clinical services in the emergency, out-patients, in-patients and Laboratory departments. The health workers included in the study were nurses, midwives, clinical officers, laboratory staff, and medical doctors.

The sample size was calculated under the following conditions: With standard normal deviation at 95% confidence interval (Z = 1.96), P-expected proportion of health workers expected to perceive positive work-life balance in rural settings in Uganda is not known. We assumed a proportion of 50% [12] and sampling error (δ) of 5%, a total of 384 health workers was considered for the study.

### Eligibility

#### Inclusion criteria

All health workers employed in the three hospitals and working in the clinical departments of emergency, outpatients, inpatients and laboratory services. All doctors, nurses, midwives and laboratory staff employed as full-time staff and fully registered by their respective professional and regulatory bodies in Uganda.

#### Exclusion criteria

We excluded all visiting health workers because of their obvious flexibility in their work and non-structed health worker/patient contact. We also excluded intern doctors, nurse, midwife, clinical officers in their practical placements. The specialized medical professionals were also excluded because of their limited numbers and availability for the study.

### Sampling procedure

The three hospitals of Gulu regional Referral, Lacor and Gulu Independent hospitals were purposively sampled because of the relatively high patient numbers. We assumed that higher patient numbers allow adequate health workers and patient engagement. Since the population of health workers in the three hospitals was accessible, we conducted a simple random sampling of 384 respondents a lottery method [27].

### Measurement of the study variables

The dependent variable, perceived WLB was measured using three parameters; perceived time, involvement, and satisfaction with work and non-work activities. The questions exploring the individuals’ subjective perception of balance between their work and other aspects of their lives included asking the health workers to state their agreement (I agree vs. I don’t agree) [20, 28] using three statements ‘ I feel that the time balance between my work and non-work is satisfactory; I feel the level involvement in my work and non-work activities is balanced; and I am satisfied with my involvement in my work and non-work activities. All the responses where the respondents ‘agreed’ with the statement were combined to composite ‘yes’ response (indicating a positive balance) which scored ‘1’ and those who did not agree where composed into ‘No’ (indicating a negative balance) which scored ‘0’ For the purposes of analysis, the dependent variable (PWLB) was dichotomized into binary outcomes. A positive PWLB = 1 and negative work-life balance = 0 [23].

The independent variables explored were the individual, health facility and community level factors assumed to be associated with the dependent variable. Individual level factors included demographics, profession duration in service, type of family, number of dependents, job satisfaction and the perception of the burden of workload. Health facility level factors included department or duty station, place of residence, duty shift (day, evening, night), time spent on duty, perception of the adequacy of staffing level, and availability of team building activities. Whereas community level factors comprised of participants’ involvement in community activities such membership in an association, community roles, and available schedule of community activities.

### Data collection tool and approach

A self-administered semi-structured questionnaire (Appendix 1) developed from literature related to work-life balance [8, 18, 20, 23, 26]. The assessment of perceived work-life balance (WLB) was composed of three statement-based items that necessitated a response. The items were ‘ I feel that the time balance between my work and non-work is satisfactory; ‘I feel that the level of involvement in my work and non-work activities is balanced’; and I am satisfied with my level of involvement and time balance of my work life and non-work activities. The participants were prompted to reflect on their professional work and non-work activities sharing their agreement or disagreement with the statements. The three-item statements of perceived work-life balance measure was tested internal consistency using Cronbach’s alpha which gave a score of 0.89 (Av. Inter item covariance = 0.178; number of items = 3; Scale reliability coefficient = 0.89).

The independent variable questions of age, gender, marital status, religion, type of family, number of dependents, profession, job tenure, residency, duty station and shift were also included.

and was pilot tested for feasibility and interpretation with 10 health workers in a hospital outside the study area. Data obtained from the pilot study was validated with the same study group after the tool was then revised [21]. The revised tool was then applied to the study participants using both physical delivery to the selected health workers and delivery by email for those who could not be found at the work place.

### Data management and analysis

Data were entered and analyzed using STATA version 15 (*STATA Corporation, College Station, TX*). Descriptive statistics were used to present the frequency of the respondents’ characteristics, and to estimate the prevalence of the outcome. Using a modified Poisson regression analysis, bivariable analysis was carried out to assesses the relationship between PWLB and each independent variable one by one. Using a forward multivariable regression with robust standard variables errors, variables with *p*-value less than 0.2 [29] were analyzed to identify the factors independently associated with PWLB. Statistical significance at this level of analysis was reported with adjusted prevalence ratios (APR) with *p* < 0.05 with their corresponding confidence intervals at 95%.

### Ethical approval and consent to participate in the study

Ethical approval to conduct the study was obtained from Lacor Hospital Institutional Research and Ethics Committee (LHIREC), Gulu (LHIREC/006/06/2021). Administrative approval was obtained from Uganda Martyrs University and Gulu District Local through the institutional and local research approvals. Further permission was sought from the hospitals that management of the hospitals that participated in the study. Voluntary informed written consent was obtained from participants of consenting age, and informed consent for participants of non-consent age was obtained from their legal guardians and assent was obtained from each of them before data collection.

## Results

A total of 384 healthcare workers participated in the study. The majority of the health-workers were females 207 (53.9%), 149 (38.8%) had 10 or more dependents in the home (dependents include children under care, relatives or any other individuals for whom the respondent held responsibility), and between the age category 20 to 40 years, 298 (77.6%), 214(56.3%) were officially married. A total of 209 (54.4%) had worked for 5–6 years prior to data collection, 157 (40.9%) worked evening shift, (Nurses (95) constituted, (42.7%), 164(42.7%) were satisfied with their job. The individual, health facility and community level related characteristics of the respondents are presented in Table 1.

**Table 1:** showing socio demographic characteristics of the pregnant women sampled (*N* = 384)

Variables (*N* = 384)	Categories	Frequency(n)	Percentage (%)
*Individual level*			
Sex	Female	207	53.9
	Male	177	46.1
Age in years	20–30	149	38.8
	31–40	149	38.8
	41–50	63	16.4
	More than 50	23	6.0
Religion	Catholic	186	48.4
	Moslem	79	20.6
	Anglican/Pentecostal	119	31.0
Marital status	Single	144	37.7
	Divorced/separated	24	6.2
	Married	216	56.3
Profession	Medical officer	72	18.8
	Clinical officer	66	17.2
	Nurse	95	24.7
	Midwife	77	20.1
	Laboratory staff	74	19.2
Duration in service (Job tenure)	Less than 1 year	43	11.2
	1–4 years	91	23.7
	5–9 years	209	54.4
	10 and more years	41	10.7
Number of dependents	1–4	112	29.2
	5–9	123	32.0
	10 and more	149	38.8

### Type of profession and perceived work-life balance (PWLB)

The study observed that the majority of the doctors, agreed to time balance 46/72(63.9%) and satisfaction balance 49(68.1). More than half 38/66(57.6%) of the clinical officers perceived time balance of work. Observation from the laboratory staff (*n* = 74) indicated agreement with time balance of 42 (56.8%), involvement balance of 42 (56.8%), and satisfaction balance 37 (50.0%). The nurses 61/95 (64.2%) perceived to a time balance, while 49/77 (64.5%) perceived time balance. The results are presented in Table 2.

**Table 2:** shows agreement with the domains of work-life balance

Profession	“I feel that the time balance between my work and non-work is satisfactory”		“I feel that the level of involvement in my work and non-work activities is balanced”		“I am satisfied with my level of involvement and time balance of my work life and non-work activities”	
	I agree	I don’t agree	I agree	I don’t agree	I agree	I don’t agree
	Freq (%)	Freq (%)	Freq (%)	Freq (%)	Freq (%M)	Freq (%)
Doctors	46 (63.9)	26(36.1)	22 (30.6)	50(69.4)	49 (68.1)	23(31.9)
Clinical Officers	38 (57.6%)	28(42.4)	22 (33.3)	44(66.7)	43 (65.2)	23(34.8)
Laboratory staff	42 (56.8%)	32(43.2)	42 (56.8)	32(43.2)	37 (50.0)	37(50.0)
Nurse	61 (64.2)	34(35.8)	58 (61.1)	37(38.9)	49 (51.6)	46(48.4)
Midwife	49 (64.5)	28(36.3)	37 (48.1)	40(51.9)	42 (54.6)	35(45.4)

### Overall PWLB

Overall, 157/384 (40.9%) of study participants reported a positive perceived WLB. The positive PWLB was scored among nurses 57/157(13%), laboratory staff 35 (9.1%). Midwives 31 (8.1%), clinical officer 22 (5.7), and medical doctors 19 (5.0%). These results are presented in Table 3.

**Table 3:** shows overall PWLB

Profession	Positive PWLB	
	Yes, n (%)	No, n(%)
Medical doctors	19 (5.0)	53 (13.8)
Clinical officers	22 (5.7)	44 (11.5)
Laboratory staff	35 (9.1)	39 (10.1)
Nurses	50 (13.0)	45 (11.7)
Midwives	31 (8.1)	46 (12.0)
**Total**	**157 (40.9)**	**227 (59.1)**

Legend- PWLB-perceived work-life balance

### Factors associated with PWLB

At bivariate level analysis there was significant statistical association between age, type of profession, number of dependents in the household, job tenure, job satisfaction, flexibility in scheduling the duty shifts, belonging to a community association and PWLB. The unadjusted prevalent ratios (UPR) with the corresponding *p*-values are shown in Table 4.

At multivariable logistic level analysis (Table 4), health workers had a job tenure of 1–4 years had 37% lower prevalence of reporting a positive PWLB (APR = 0.63, 95%CI: 0.40–0.99, *p* = 0.047) compared to those that has worked for less than one year. Laboratory staff had 1.7 times increased chances of positive PWLB (APR = 1.74, 95%CI: 1.10–2.75, *p* = 0.018), midwives had 1.8 chances (APR = 1.82, 95% CI: 1.13–2.93, *p* = 0.014, and nurses were 2.19 times more likely to report a positive PWLB (APR = 2.19, 95% 1.45–3.30, *p* < 0.001) compared to the medical doctors. Similarly working in the inpatient departments (AOR = 1.97, 95%CI: 1.31–2.96, *p* = 0.001, and laboratory (APR = 2.09, 95%CI: 1.34–3.28, *p* = 0.001), increased chances for reporting a positive PWLB compared with working in.

**Table 4:** showing the bivariable and multivariable logistic regression analysis of PWLB among respondents

Variable Category	PWLB		UPR (95% CI)	*p*-value	APR (95% CI)	*P*-value
	No (%) *n* = 126	Yes (%) *n* = 156				
**Age (in years)**						
20–30	98(43.2)	51(32.5)	1		1	
31–40	81(35.7)	68(43.3)	1.33(1.004–1.77)	**0.045**	1.10(0.72–1.68)	0.672
41–50	36(15.9)	27(17.2)	1.44(0.78–2.63)	0.224	1.11(0.70–1.76)	0.653
> 50	12(5.2)	11(7.0)	1.76(0.79–4.27)	0.174	1.19(0.58–2.48)	0.634
**Religion**						
Catholic	120(52.9)	66(42.0)	1		1	
Anglican	65(28.6)	54(34.4)	1.28(0.97–1.68)	0.082	1.22(0.91–1.63)	0.060
Muslim	42(18.5)	37(23.6)	1.60(0.94–2.73)	0.074	1.33(0.97–1.84)	0.076
**No. of dependents**						
< 5	51(22.5)	49(31.2)	1		1	
5–9	85(37.4)	49(31.2)	0.75(0.55–1.02)	**0.056**	0.78(0.57–1.06)	0.117
≥ 10	91(40.1)	59(37.6)	0.80(0.61–1.05)	**0.127**	0.89(0.67–1.19)	0.447
Gender						
Female	126(55.5)	81(51.6)	1			
Male	101(44.5)	76(48.4)	1.10(0.80–1.50)			
**Job tenure (years)**						
> 1	23(10.1)	20(12.7)	1		1	
1–4	64(28.2)	27(17.2)	0.63(0.41–1.01)	**0.051**	0.63(0.40–0.99)	**0.047**
5–9	120(52.9)	89(56.7)	0.92(0.64–1.30)	0.629	0.79(0.55–1.12)	0.183
≥ 10	20(8.8)	21(13.4)	1.10(0.71–1.71)	0.667	1.02(0.64–1.59)	0.942
**Profession**						
Doctor	53(23.4)	19(12.1)	1			
Laboratory staff	39(17.2)	35(22.3)	1.79(1.14–2.83)	**0.012**	1.74(1.10–2.75)	**0.018**
Midwife	46(20.3)	31(19.8)	1.88(0.94–3.76)	**0.080**	1.82(1.13–2.93)	**0.014**
Nurse	45(19.7)	50(31.8)	1.53(0.95–2.44)	**0.002**	2.19(1.45–3.30)	**< 0.001**
Clinical officer	44(19.4)	22(14.0)	1.26(0.75–2.11)	0.375	1.23(0.74–2.06)	0.429
**Duty station**						
Emergency	68(30.0)	25(15.9)	1		1	
Inpatient	44(19.4)	41(26.1)	1.79(1.20–2.68)	**0.004**	1.97(1.31–2.96)	**0.001**
Laboratory	37(16.3)	45(28.7)	2.04(1.38–3.01)	**< 0.001**	2.09(1.34–3.28)	**0.001**
Outpatient	78(35.3)	46(29.3)	1.38(0.92–2.07)	**0.121**	1.39(0.92–2.08)	0.117
**Duty shift**						
Day	90(39.6)	43(27.4)	1		1	
Evening	86(37.9)	71(45.2)	1.40(1.04–1.89)	**0.029**	0.83(0.71–0.98)	**0.029**
Night	51(22.5)	43(27.4)	1.41(1.02–1.97)	**0.040**	0.96(0.80–1.17)	0.751
**Satisfied with work**						
Not satisfied	42(18.5)	122(77.7)	1		1	
Satisfied	185(81.5)	35(22.3)	0.21(0.16–0.29)	< 0.001	1.58(1.17–2.10)	**0.001**
**Flexible schedule**						
No	224(98.6)	8(5.1)	1			
Yes	3(1.4)	149(94.9)	28.42(14.37–56.23)	**< 0.001**	28.32(14.52–55.22)	**< 0.001**
**Belongs to the community association**						
No	199(87.7)	4(2.6)	1			
Yes	28(12.3)	153(97.4)	42.89(16.20-113.56)	**< 0.001**	32.71(11.91–89.88)	**< 0.001**

Legend

**Bold** – statistically significant, PWLB-Perceived work-life balance, UPR-Unadjusted prevalence ratios, APR-Adjusted prevalence ratios

emergency department. Also, health worked that expressed job satisfaction, (APR = 1.58, 95%CI: 1.17–2.10, *p* = 0.001) were 1.58 more likely to report positive PWLB while those who had worked evening shift (APR = 0.83, 95% CI: 0.71–0.98) were 17% less likely to perceive positive work-life balance. Health workers that experienced flexibility in the scheduling of their duty shifts (APR = 28.32, 95% CI: 14.52–55.22, *p* < 0.001) and those who belonged to any community association were (APR = 32.71, 95% CI: 11.91–89.88, *p* < 0.001) were 28.3 times and 32.7 times likely to report positive PWLB compared to those who did not have similar experiences.

## Discussion

Balancing between work, non-work and family demands is a challenge in many professions [24]. In this study four in every 10 health workers reported that they are not able to balance their work (patient care) with their important non-work and community roles. This implies a negative effect on their personal, professional [17] and social belongingness [19]. Workers are often happier after they are able to balance their work demands and are able to sustain their important family and community obligations [12]. It suffices to note that every individual person’s circumstance or current situation may affect how he/she perceives the extent to which their work, non-work or family is central to their life existence and so may affect their perception of work-life balance [24], an indication that the feeling of positive balance is likely to change in difference circumstances. However, the results are almost similar studies [17, 18] where less than half of the health workers experienced difficulties in maintaining their work-life balance. Other studies [10, 15] where health care organization had WLB strategies, the results for WLB were satisfactory and respondents were mostly satisfied with WLB. This implies in situations where deliberate effort if put into strategies that support PWLB health workers may feel a reasonably comfortable balance between work and non-work commitments.

In this study factors associated with perceived work-life balance assessed from individual, health facility and community level attributes indicated that at individual level, the profession of laboratory, midwifery and nursing had a positive likelihood of balancing work and non-work roles. This is contradicts other studies [16, 17] where nurses in general experience high levels of burn out consequent to negative PWLB. By and large, the departments where these workers are assigned roles which offer supplementary roles or supportive roles to patient care, may not feel the heavy burden of workload [24] and may have higher likelihood of positive PWLB. On the other hand, having number of dependents (5–9 people) was associated with a strain on the perceived work-life balance. This is similar to finding from a systematic review and another research carried out among early career pediatricians [18, 24]. Under such circumstances work-family conflict that arise creates a situation having ‘much to do’ or role overload [24] especially when spending more time at home means cutting off some of the time for social and community activities. Other studies carried especially during COVID-19 [4] relationship such spouse/ dependents/ family were positively associated with WLB. This could imply that the restrictions caused by COVID-19 that allowed work from home allowed exploration of the supportive role of relationships. Job satisfaction was positively associated with WLB a situation that is similar other studies [18] that showed a positive relation between career satisfaction with work-life balance. Analysis of gender (comparing female and males) did not yield statistical significance in this study which similar to a study conducted in Uganda among nurses [16], in another study carried out among early career pediatricians [20], female early career pediatricians were associated with lower perceived work-life balance.

At health level or setting level flexibility in scheduling of work [25]; were positively associated with work-life balance. This means that health workers that worked in the evening and also allowing a level of flexibility enhances job control [26] which in many cases relates positively with the way health workers perceive the balance of their work and other activities. Allowing a reasonable personal and professional comfort at work through proper scheduling of work that allows meaningful family and community responsibilities [12] enhances the quality of work-life in turn helps works to create and maintain positive work-life balance [1].

Belonging to a social association was associated with increased likelihood for positive work-life balance probably due to an inherent ability of such a relationships to offer the needed social support [21, 30] worker need. Assigned community roles such church activities were associated with reduced likelihood for a positive WLB because such activities tend increase role overload [24, 31] which does not favor work life balance.

The study has some limitations. A cross-sectional study takes measurements at a point in time, a weakness in estimating causality. However, with relatively big sample estimation of the association between the outcome variable and factors is acceptable. Secondly, we used self-reporting without structured work-life balance scale. This means that stated perceived measure of balance for one individual could be different for another. However, this acceptable since the study adopted individualized perception of WLB. Lastly, in the study, we sampled more medical doctors than what is in the health facilities in Gulu. This was because data was collected during the COVID-19 restrictions that allowed only essential works in the health facilities exaggerating the numbers of medical doctors.

## Conclusion

Less than half of the health workers experienced perceive positive work-life balance and this was associated with the type of profession with mainly supportive roles for patient care, the number of dependents, nature of the shift, scheduling of work and availability of support systems. On the other hand, role overload was associated with less likelihood of positive WLB balance. Therefore, nurturing a system of reviews of the scheduling of health workers and fostering support systems around the health workers is very beneficial for WLB. This information will help health care managers and their employers to design WLB strategies to improve health workers improve their perception of how they balance work and non-work roles and commitments.

### Electronic supplementary material

Below is the link to the electronic supplementary material.

**Supplementary Material 1: Appendix 1:** Questionnaire for factors associated with perceived work-life balance

## Data Availability

All relevant data are included in the paper. The questionnaire has been included as a supplementary material.

## Abbreviations

APR: Adjusted Prevalent Ratio
PWLB: Perceived Work Life Balance
UPR: Unadjusted Prevalent ratio
WLB: Work Life Balance
