# Supplementary material for: Factors associated with perceived work-life balance among health workers in Gulu District, Northern Uganda: a health facility-based cross-sectional study
Source: BMC Public Health. 2024 Jan 23;24:278. doi: 10.1186/s12889-024-17776-8 (PMC10807176; doi:10.1186/s12889-024-17776-8)
Supplement: Supplementary file 1 — Supplementary Material 1: Appendix 1: Questionnaire for factors associated with perceived work-life balance [file 12889_2024_17776_MOESM1_ESM.docx]

# **APPENDIX 1: QUESTIONNAIRE FOR FACTORS ASSOCIATED WITH PERCEIVED WORK-LIFE BALANCE**

**Section A: Socio-demographic characteristics of the health workers**

| **Number** | **Question** | **Choices** |
| --- | --- | --- |
| 1 | Age in full years | ……………………………… |
| 2 | What is your current marital status? | Married❒ Single ❒ Divorced/Separated❒ Other ❒ |
| 3 | To what religious denomination do you belong? | Catholic ❒ Anglican ❒ Muslim ❒ Other……… |
| 4 | How many dependents do you stay with at home? | None ❒ 1-4 dependents ❒ 5-9 dependents ❒ 10 and more ❒ |
| 5 | What kind of family do you have? | Nuclear family ❒ Extended family ❒ |
| 7 | Where do you stay while working in the hospital? | Stay in the hospital owned staff houses within the hospital premises ❒ stay outside the hospital premises |
| 8 | What is your professional cadre? | Medical Doctor ❒ Nurse ❒ Midwife❒ clinical office ❒ Laboratory Technician/assistant ❒ |
| 7 | How long have worked in this health facility? (Job tenure) | Less than 1 year ❒ 1-4 years ❒ 5-9 years ❒ ≥ 10 years |
| 8 | How satisfied are you with your current job | Satisfied ❒ Not satisfied ❒ |

**Section B: Work-life balance**

|  | **Response item** | **Choices** |
| --- | --- | --- |
| **9** | ‘I feel that the time balance between my work and non-work is satisfactory’ | I agree ❒ I don’t agree ❒ |
| **10** | ‘I feel that the level of involvement in my work and non-work activities is balanced’ | I agree ❒ I don’t agree ❒ |
| **11** | ‘I am satisfied with my level of involvement and time balance of my work life and non-work activities | I agree ❒ I don’t agree ❒ |
| **12** | Under the currently existing condition in my health facility, I feel I am able to balance my work, non-work, and personal life obligations. | I agree ❒ I don’t agree ❒ |

**Section C: Health facility-related factors**

|  | **Question** | **Choices** |
| --- | --- | --- |
| **13** | In which Department are you assigned clinical work in your hospital? | Outpatient department ❒ inpatient department ❒ Emergency department ❒ Laboratory department ❒ |
| **14** | What is your current duty shift scheduled for you? | Day shift ❒ Night shift ❒ Evening shift ❒ |
| **15** | What is your employment status? | Confirmed in my job ❒ Not confirmed in the job ❒ I work as a part-time employee ❒ I work as a volunteer ❒ |
| **16** | What is the length of your current duty shift | ≤ 8hours ❒ 9-12 hours ❒ > 12 hours |
| 17 | Does your health facility allow flexibility in the scheduling of day, evening, and night shifts? | Yes ❒ No ❒ |
| 18 | Do you feel that your health facility has enough staff to handle the patient load? | Yes ❒ No ❒ |
| 19 | Does your health facility support team-building activities such as shared coffee breaks? | Yes ❒ No ❒ |

**Section D: Community factors**

|  | **Question** | **Choices** |
| --- | --- | --- |
| 20 | Do you have some role(s) you perform in your community other than health service delivery? | Yes ❒ No ❒ |
| 21 | If you perform roles in the community other than service delivery, when do you attend to your community roles? | During the week (Monday - Friday ❒ During weekend days (Saturday -Sunday ❒ |
| 22 | Do you belong to any community association? | Yes ❒ No ❒ |
| 23 | Do ever engage in community church-related activities? | Yes ❒ No ❒ |
| 24 | If yes, how often do you engage in community church-related activities? | Weekly❒ Monthly ❒Annually ❒ |
